# Supporting first-time parents in their homes: an informal setting enabling interprofessional collaboration

**DOI:** 10.1186/s12913-024-10949-6

**Published:** 2024-04-29

**Authors:** Cecilia Franzén, Eva-Lotta Nilsson

**Affiliations:** 1 Faculty of Odontology, Malmö University, Malmö, Sweden; 2 Faculty of Health and Society, Malmö University, Malmö, Sweden

**Keywords:** Boundary work, Child healthcare, Dental care, Maternal care, Social services, Home visit programme, Interprofessional collaboration, Qualitative interviews

## Abstract

**Background:**

Home visiting programmes aiming to support parents and promote more equal health amongst young children have grown in Sweden and in other countries. These programmes involve interprofessional teams. Teamwork in interprofessional contexts often requires setting boundaries, but professionals’ boundary work in the home setting is unexplored. Therefore, this article focuses on interprofessional teams comprising child healthcare nurses, midwives, social workers, and dental hygienists in a home visiting programme for first-time parents in Sweden; it aims to explore how the professionals performed boundary work that enabled collaboration and to investigate important contextual conditions for this kind of boundary work.

**Methods:**

The data were drawn from semi-structured interviews with twelve professionals from the four different disciplines. Content analysis was used to explore their boundary work.

**Results:**

The findings show that the professionals performed three forms of collaborative boundary work. They maintained boundaries by clarifying their distinct roles and expertise. However, the differences were viewed as complementary, and the professionals worked together humbly to complement each other’s knowledge and perspectives. Lastly, they tended to drop perceptions of prestige and blurred the boundaries to accommodate their overlapping knowledge. Important conditions for the success of collaborative boundary work were meetings prior to the home visits, the opportunities for discussion and reflection after the home visits, and the informal character of the home setting. Consequently, the professionals were able to jointly contribute to a holistic view of the visited families, which increased the possibilities to meet these families’ needs.

**Conclusions:**

This study contributes knowledge on boundary work in interprofessional collaborations in the home setting. The informal character of the home setting seemed to facilitate collaboration and contributed to creating informal professional roles. The findings suggest that having interprofessional teams in the home setting enabled collaboration as well as reinforced support for first-time parents, which emphasizes the merit of home visit programmes.

**Supplementary Information:**

The online version contains supplementary material available at 10.1186/s12913-024-10949-6.

## Background

The need for health professionals from different disciplines to work in teams, instead of in silos, has been emphasized on both political and managerial levels to meet population needs and to improve cost-effectiveness, quality, and access to health services [1, 2]. Consequently, interprofessional teams have become more common in diverse settings, such as in hospitals [e.g. 1, 3–8] and in dental care clinics [9–11]. During the past few decades, it has become more common for professionals to work in interprofessional teams in non-clinical settings, such as in home visiting programmes for parents with young children [12, 13], in care at home for older persons with multimorbidity [14] and in advanced care at home for patients with significant healthcare needs or chronic illnesses [15].

This article focuses on interprofessional teams in a home visiting programme for first-time parents in the south of Sweden to promote more equal health amongst young children and support these parents in maintaining good health practices. Home visiting programmes for parents with young children typically utilize professionals from different disciplines, such as nurses, social workers, psychologists, physicians, and counsellors [13, 16]. The rationale behind the development of interprofessional teams in home visiting programmes is that early prevention and professional collaboration is believed to ensure children’s health and growth and improve parents’ self-efficacy and health [17]. Thus, the home visiting programme in question required that child healthcare nurses conducted home visits together with midwives, social workers, or dental hygienists/dental nurses [18]. The professionals in the home visiting programme in question had not previously worked together in the teams.

The outcome of interprofessional collaboration in healthcare might not be self-evident as professionals might have different interests, values, and views on how to take care of patients and clients [19]. These differences might cause the collaboration to be hindered or aggravated by professionals’ attempts to defend boundaries between their professions [6, 7]. On the other hand, professionals can also collaborate well by blurring and downplaying boundaries between them. Previous studies have mostly focused on boundaries in relation to collaboration in hospital or clinical settings, so there is a need for more studies on collaboration in patients’ homes [1]. Conducting home visits means changing the setting of work for healthcare professionals from formal spaces, such as hospitals, to a more informal setting [20]. The home setting should not be regarded as just an extension of a work organization but as a distinct sphere of practice and experiences in its own right [21]. That is, the home visit is conducted in a terrain that is ‘not intrinsically connected to any particular profession’ as it is away from the clinic or the office where professionals work [22, p.349]. This article contributes knowledge on interprofessional collaboration provide healthcare in the home setting by focusing on the professionals’ boundary work and its implications on their collaboration when conducting home visits with first-time parents.

The need for individuals from different professions to work together has implications for how they coordinate their work tasks and roles. Despite regulations of task division for professionals within healthcare, dental care, and social services, there is room for negotiations in daily work through professionals’ boundary work, in which they construct, maintain, blur, cross, or downplay professional boundaries [23–26]. Thus, professionals’ boundary work is significant in the understanding of interprofessional work relations [8] and it has been described as a form of “floor politics”, that is professionals’ struggles and negotiations that takes place in everyday work [3].

Professional boundaries are not static; they can be ‘understood as socially constructed demarcations that establish what is, and what is not, a profession’s sphere of competence and a legitimate domain of activity’ [6]. As boundaries are constructed, it opens for negotiations on work division and work roles at workplaces. Negotiations take place primarily during conditions of change, uncertainty, and ambiguity [23], which may occur when professionals have to work in newly created teams. When engaged in boundary work, professionals can stress both similarities and differences between themselves and members of other professions [27]. The distinction between ‘them’ and ‘us’ can be drawn in various ways depending on the individuals’ perspectives and experiences [1]. For example, healthcare professionals’ specific norms and values concerning patient care might differ from those of other professionals. Professionals can have different views on what constitutes evidence, safe practice, correct patient treatment, and high-quality patient care [7].

Professionals’ boundary work can be interpreted as competitive and collaborative. Competitive boundary work concerns how professionals construct, defend or extend boundaries to distinguish themselves from others to achieve some kind of advantage [28]. Competitive boundary work may have consequences for health professionals’ work on patients. Defence of existing professional boundaries and roles may impede meeting the complex needs of patients [6] and can hinder interprofessional collaboration for high-quality patient care [7]. Similarly, studies on the Swedish dental care [10, 11] and healthcare [29] have revealed interprofessional tensions and conflicts about work tasks which may hinder different professional groups’ skills to be utilised in the best ways in the caring of patients.

However, maintaining boundaries around a certain practice and knowledge can also be interpreted as collaborative boundary work, for example, when professionals respect their different niches of expertise [30] and when they appreciate their different contributions in the care of patients [1]. Collaborative boundary work also refers to professionals’ negotiation, blurring or downplaying of boundaries in interaction with others in order to collaborate to get their work done [28]. For example, nurses’ role may require negotiations over performing tasks in a hospital context, and nurses can cross boundaries and undertake doctors’ tasks based on assumptions about what is best for the patients and based on the nurses’ aspirations to provide good care [3].

Summing up, families’ home is a place that is described as more informal compared to health professionals’ workplaces and as a place that doesn’t belong to any specific profession. This gives rise to the question of the significance of the families’ home as a specific setting for interprofessional collaboration and boundary work. This article demonstrates how the professionals in the home visiting programme performed boundary work that enabled collaboration.

### Research context

Child healthcare in Sweden is a service that is offered free of charge to all children up to the age of five years. The service aims to promote children’s health and development, to prevent disease, and to initiate inventions when needed. All families are offered home visits by a child healthcare nurse at one to two weeks and at eight months after birth. In recent, there has been a development of extended home visiting programmes conducted by interprofessional teams. This development is a result of funds from the Swedish government to promote more equity in health and to meet the needs of families with young children [31].

The home visiting programme in question was supported by politicians at the regional level. The project ran from September 2019 until the end of 2022. The project started with four teams in 2019; thereafter, twenty-four teams joined, but two of these dropped out. The programme entailed a collaboration between professionals from child healthcare, maternal care, social services, and dental care, who worked in teams and visited first-time parents in their homes. The programme included six home visits during a child’s first 15 months. The home visits were carried out by a child healthcare nurse together with a midwife (1–2 weeks after birth), a dental hygienist or a dental nurse (8 months after birth), or a social worker (2–3 weeks then 4, 10, and 15 months after birth). The home visits focused, for example, on breastfeeding, parenthood, the health of the child, and good oral health habits. The professionals had guidelines for each home visit but some freedom to meet the needs of the visited parents and children and adapt their information and support.

## Methods

### Data collection

This article draws on a larger research project concerning managers and professionals of the four initial teams that participated in the home visiting programme starting in September 2019. The empirical material in this article was based on semi-structured interviews with professionals from these four teams. 12 of totally 15 professionals agreed to be interviewed: four child healthcare nurses (CN), two midwives (MW), three dental hygienists (DH), and three social workers (SW). There were only three dental hygienists as one participated in two of the four teams. It was not possible for two midwives and one social worker to participate in this study.

The interviews were conducted by three researchers, two of them are the authors of this article. The professionals were interviewed individually and digitally from November 2020 to May 2021 due to the COVID-19 pandemic. The interviews ranged from 45 to 60 min, and they were recorded and then transcribed by the authors of this article. The interviews were semi-structured with room for the participants to reflect on their experiences [32]. The interview guide can be found in additional file 1. The interview questions that are of relevance for this article concerned (a) the professionals’ experiences of the home visiting programme and of conducting home visits together with other professionals and (b) their views on their own and the other professionals’ roles and expertise. The interview guide has previously been used in a study on dental professionals’ participation in the home visiting programme. The interview questions were based on the literature and the researchers’ theoretical knowledge of interprofessional collaboration, preventive work for improved health and wellbeing, and political support for equal health [33]. Prior to the interviews, the researchers emailed the professionals the aim of the study, the interview questions, and an invitation to participate. Further, written informed consent from the professionals was obtained before the interviews. This study has been ethically assessed by the Swedish Ethical Review Authority (dnr. 2020–03435).

### Data analysis

The interviews were analysed using content analysis with the aim of classifying the responses into categories and then into overarching themes [34]. In the analysis, the first author initially read the transcribed interviews thoroughly several times to get an overview and to identify data relevant for the aim of this article– namely, data interpreted as relevant to understand what types of boundary work the professionals performed or how they performed boundary work. This analytical process comprised repeated reading of both the empirical data and the literature on professional boundary work. This process contributed to a renewed interpretation of the selected data. The relevant data were grouped into categories that were then grouped into themes through a comparison between data that belong to a theme and others that do not belong to it. This culminated in three different kinds of boundary work: maintained, downplayed, and blurred boundaries. Thereafter, both authors jointly discussed the derived themes. Finally, we reanalysed the data to interpret contextual conditions that enabled the collaborative boundary work. The themes and the categories can be seen in Table 1.

**Table 1:** The derived themes and categories

Themes	Categories
Maintained boundaries	Specific expertise and roles
	Differences between the professionals
	Clear roles
Downplayed boundaries	Complementary expertise and roles
	Benefits of working together
	Humble collaboration
Blurred boundaries	Fluid conversations
	Overlapping knowledge
	No prestige
Conditions for collaboration	Opportunities for professional meetings before home visits
	Time for reflections before and after the home visits
	The home setting

## Results

### The professionals’ boundary work

The analysis revealed three themes that demonstrated the professionals’ boundary work: (1) maintained boundaries– clear professional roles and expertise, (2) downplayed boundaries– humble and complementary collaboration, and (3) blurred boundaries– informal roles and collaboration. The themes will be presented below. The quotes that we present are used as illustrations of the content of the themes.

#### Maintained boundaries– clear professional roles and expertise

The professionals in the teams reflected on both their own and others’ roles, expertise, and perspectives in the home visits. The child healthcare nurses worked with members of all the other three professions, thereby allowing them to make comparisons with their professional counterparts and vice versa.

The expertise and roles that clearly differed were those of the midwives and child healthcare nurses. Both the midwives and the nurses described the midwives’ work during the home visits as focusing on the women, particularly their experiences of delivery and their well-being the first weeks thereafter. They could also extend their concern to the women’s partners. One midwife explained,

As a midwife, I am interested in how they [the women] experienced the delivery and their body, whether it works well physically but also how they handled the experience of the delivery. My focus is on the woman and on the partner. The focus of the child healthcare nurses is more on the children. (MW1)

Another difference related by a midwife was that child healthcare nurses ‘are better with breast-feeding’ (MW2). One child healthcare nurse similarly separated the roles of the two and indicated the value of the differences for the collaboration:

The midwife became a natural [partner] as she has the delivery and the pregnancy to talk about, and we focus on the children and their health. (CN3)

Other child healthcare nurses also stressed that their focus was on the children, that they have more specific knowledge of children compared to the other professionals, and that they have to carry out special development checks. Child healthcare nurses further related that because they participated in all the six home visits, they thus had an overarching responsibility for the families’ well-being and support needs.

Dental hygienists conducted home visits together with child healthcare nurses when the children were eight months old. Undertaking a dental and oral examination for young children is under the auspices of both the dental professionals and the child healthcare nurses. Therefore, although the child healthcare nurses are not educated in dental care, they have some knowledge in the area. This was alluded to by one of the dental hygienists: ‘She does not have a dental education, but still, they know a little about teeth’ (DH1). However, there are differences between the two sets of professionals regarding dental knowledge. One child healthcare nurse reflected on her own shortcomings:

We talk a lot about teeth. And we talk about good eating habits and tooth brushing during almost every visit. And we examine the children’s mouth. So I thought I was pretty good at that. But when I began to work with the dental hygienist, the discussion became so much better, and I have noted how perfunctory I have been compared to her. (CN2)

The gap in dental knowledge was further emphasized by another dental hygienist when comparing the two professions:

We have pretty specific knowledge about dental health, which strictly speaking is not so remarkable, but it is still beyond their main focus area. (DH2)

Both the social workers and the child healthcare nurses focus on parenting and the relationship and attachment between parents and children. The highlighted differences between the two professions concerned the depth of conversations with the parents on these aspects:

We have the similar values. They [the child healthcare nurses] talk a lot about parenting and interaction and attachment. We probably have more in-depth knowledge, however. (SW1)

The roles within the teams were described as both clear and divided. According to the professionals, having separate roles and offering different contributions were factors that facilitated a successful collaboration. A child healthcare nurse exemplified this in her remarks on work in the newly created teams:

One should be clear [about the following]: what can we offer? what can we not offer? what is my role? what is your role? And even if we work in a team, we have different roles. This must be clarified. (CN4)

To sum up, the distinct professionals’ expertise and roles were described as a prerequisite for collaboration to work. However, as we will show below, the boundaries were not viewed as obstacles to collaboration, and they could be downplayed and blurred during the home visits.

#### Downplayed boundaries– humble and complementary collaboration

Due to their different knowledge and perspectives on children’s health and parenting, the professionals viewed the support they provided for the families as complementary. They also described the relationship between them as humble. For instance, they talked about the benefit of ‘seeing things through different perspectives’ and ‘having a different set of eyes when we look at things’. Furthermore, they explained how their different knowledge and perspectives could result in better discussions with the visited families compared to child healthcare nurses meeting families on their own.

Child healthcare nurses related that they worked in a complementary relationship with the others. They described the knowledge and role of the midwives, who focus on the mothers’ wellbeing pre- and postpartum, as clearly different from their own. Thus, both sets of professionals could jointly support the new mothers. A midwife conveyed a similar view:

The child healthcare nurse and I have addressed some upcoming questions. If she was unable to provide an answer, I was able to intervene, so it has worked very well. She knows more about breastfeeding, while I know more about childbirth. We have complemented [each other]. (MW2)

Further, a child healthcare nurse and a social worker stated that working in a pair at the home visits led to better discussions with the parents:

The social workers have a lot of experiences and knowledge. I do not want the informative role, but I strive for conversation, a discussion with the family. And I find it much easier when there are more people. (CN1)

We might have a slightly different focus at the home visits, but they can be complementary. Needless to say, the conversations will be good. (SW1)

The complementary focus of child healthcare nurses and dental hygienists was also valued by both parties, particularly the shared knowledge on children’s oral health. A child healthcare nurse elaborated on how rewarding it was to work together with a dental hygienist:

I love to collaborate. I think this makes us more capable than working alone. I didn’t know how it would be [working with a dental hygienist]. In hindsight, I think this [collaboration] has been the best with regards to how much we can give, help, and support each other. (CN2)

The benefits of collaborating with child healthcare nurses in terms of providing supplementary support was also highlighted by the dental hygienist, who described the relationship as imbued with humility:

We complement each other quite a lot. We greatly help each other with tips on how and when to brush [teeth] and on breast-feeding. So, we have not disagreed at all. We are pretty humble with each other. (DH3)

As evident from the above examples, the participants acknowledged the benefits of their complementary collaboration on the visited families. The different professionals could jointly contribute to a more comprehensive view of what constitutes being parents of young children and catering for their needs. Additionally, when two different professionals viewed an issue from a different position and could still reach agreement, this served to emphasize the message to parents.

#### Blurred boundaries– informal roles and collaboration

The communication between the professionals seemed to be informal, and they did not always separate their roles when meeting the parents. Consequently, the conversations were fluid, with the professionals sometimes interjecting when necessary, thus implying their roles could be blurred. Social workers related that the blurring of roles during home visits was due to the partly overlapping knowledge and focus between them and the child healthcare nurses. One of them explained:

Much of what we discuss lies in a grey zone, in which we can take turns. That isn’t something we preplan. Instead, it depends on the situation, on how the discussion unfolds. So, it has not been a big deal for us but has felt natural. (SW3)

The importance of regulating who should say what and when was also experienced by dental hygienists and child healthcare nurses. The division of roles and conversations occasionally became more blurred when the professionals had worked together for a while and had gotten to know each other. A child healthcare nurse reflected on her relationship with the social worker and the dental hygienist during home visits and described how the conversations with the parents became blurred:

The longer we have worked together, I think the more we don’t have any clear roles anymore. There has not been any sense of prestige with either of us. Instead, the families’ best has really been the focus. (CN2)

Working in teams without prestige and prioritizing the best of the families were also highlighted by a dental hygienist. Importance was not placed on any division of roles in the conversation but on the parents getting the required information:

The collaboration with the child healthcare nurses became immediately easy, without any prestige. Nobody was protective over what to say: ‘I have to say that. That is mine.’ Instead, we would talk together. If the child healthcare nurse happens to inform [the parents] about everything, I don’t take offence. I don’t intersect just to outshine or say a lot but rather to do what’s best for the family. (DH2)

As the quotations show, the professionals valued the lack of prestige between them. They also appreciated that the way they should talk to parents was not decided in advance. Fluid roles were believed to contribute to successful collaboration and to discussions with parents that were grounded in each family’s specific needs.

### Conditions for collaboration

Based on the interviews with the professionals, we found three important conditions for boundary work that led to good collaboration: professional meetings before the home visits, time for reflections before and after the home visits, and the home setting itself.

#### Professional meetings before the first home visits

The professionals stressed the importance of getting to know each other before they conducted the home visits. For instance, to present a united front at the meetings with the parents and their children, one team arranged preparatory meetings before the programme started to discuss possible conflicts of values and knowledge between the involved professionals.

The whole team met and discussed different scenarios before we went out. What is important for you and what is important for me? (CN2)

#### Time for reflections before and after the home visits

Some of the professionals used the time before and after the home visits for informal meetings. When they met outside a family’s home, they would talk on the phone before the home visit, and when they walked or went by car together, they used this time to talk to each other. These conversations concerned the family they were visiting and their experiences of the meetings with the family. For example, one child healthcare nurse described this kind of reflection as an opportunity to broaden her perspective on how to support parents and to learn about the others’ perspectives:

I reflect together with my colleague who I have been on a home visit with, ‘What did you think? I saw this.’ (CN2).

Professionals could also discuss what happened after a home visit. Thus, an important condition for successful collaboration was time for the professionals to talk to each other.

#### The home setting

The findings indicate that the professional roles and boundaries were blurred and downplayed during the home visits. The home setting appears to be an important condition for such boundary work, due to its relaxed and informal nature. For instance, the participants stressed that when they met the families in their own homes, the parents and the child were comfortable and relaxed:

Many [families] become more relaxed in their own environment. (SW2)

The home setting was also described as comfortable for the professionals, influencing how they acted during the home visits. When reflecting on the home visits, one dental hygienist stressed the relaxed relationships between the professionals and between the professionals and the families during a home visit compared to interactions at the professionals’ workplace:

It is more personal, and the children are usually very relaxed in their own environment. It is a very relaxed environment, and it is not as formal as it is when one is at the clinic. We sit on the floor on the same level as the child and talk, very laidback. (DH2)

This implies the contribution of the relaxed and impersonal environment for boundary work. That is, it facilitated the blurring or downplaying of boundaries, which in turn led to a collaboration that was characterized by humility and by lack of prestige and hindering boundaries.

## Discussion

Professional boundaries are important for how health professionals operate and work together [7], but little is known about collaborative boundary work in the home setting [1]. In this article, we have analysed interprofessional collaboration in newly created teams in a home visiting programme for first-time parents. Our findings show that the professionals experienced having clear and distinct professional knowledge and roles. However, although differences in expertise and responsibilities were emphasized, they were not regarded as a catalyst for conflicts or barriers, as they might be when professionals work together in new teams [35]. Rather, the professionals’ collaboration was characterized by downplayed and blurred boundaries, humility with each other, and lack of prestige regarding their own roles and expertise. Accordingly, this study is in line with the contention that the increasing demand for health professionals to work together does not have to be burdensome for the professionals [36], and that professionals’ boundary work does not have to be competitive but can be collaborative [28, 30].

The success of the interprofessional collaboration explored in this study may be attributed to several factors. One reason can be that it took place in the home setting. The home is an informal environment compared to hospitals and other formal institutional settings [20]. Consequently, the interactions between the professionals and between the professionals and the visited families were also informal. That is, the informality of the setting seemed to have a positive influence on the collaboration and relationships between the professionals. The home is also a work environment not belonging to any particular profession [22]. As indicated in this study, the home setting reduced the professionals’ territory and claim over a particular role when they met the families. Further, the opportunities for the professionals to reflect on their own and the others’ roles likely played a role. Thus, the home setting and the opportunities for meetings before and after the home visits were important contextual conditions that underscore the merit of conducting home visits.

Still another reason for the good collaboration could be that the professionals worked for the best of the families. The professional differences were stressed as significant in supporting the families because the team members’ knowledge and perspectives on children’s development and parenting complemented each other, which in turn enabled a more holistic approach to family needs. The results are in line with previous studies which showed that the maintenance of disciplinary boundaries can contribute to collaboration when professionals perceive their specific and different knowledge as necessary for the good care of patients [1] and when they respect the differences between themselves and others [30]. It may also be the case that the professionals in this article contributed to a successful collaboration through blurring and downplaying boundaries because they worked towards the shared goals of meeting the families’ needs and supporting the parents in the best possible ways. A study in a hospital setting showed that different health professionals together acted in what they believed to be the best interest of the patients and were driven by an ideology of caring [3]. Consequently, the expectation of the professionals in the home visiting programme to collectively support parents in their parenting, which was set by political intentions [18], seemed to be realized.

The successful collaboration could also be the result of all the different professionals being members of semi-professions, so-called welfare professions. Such professions have shorter university training and a lower status compared to physicians, dentists, and lawyers, and other high-status professions [37]. It has been argued that welfare professionals are likely to be more welcoming compared to high-status professionals. Welfare professionals have limited exclusive knowledge compared to their high-status counterparts, which means they set fewer or weaker boundaries around their work tasks compared to high-status professionals. Further, welfare professionals are described as embracing an ideology of person-centredness, making them more open to collaboration [38]. However, studies on the working relationship between professionals from different hierarchical levels– for example, between nurses and doctors [39] and between dentists and dental nurses [10]– have also shown blurred boundaries. Nevertheless, the types of professionals in the home visiting programme explored in this article seemed to be suited for collaboration and for support adapted to the needs of the individual families.

### Strengths and limitations of the study

This article focused on the views of midwives, child healthcare nurses, social workers, and dental professionals regarding how they worked together. This is a strength as it provided us the opportunity to describe the interprofessional collaboration from all these different professional perspectives. Moreover, the data were first interpreted by the first author and then discussed by both authors to strengthen the interpretation. One limitation of this study is that it only concerned the four initial teams in the programme. However, its explorative character was not affected negatively by the small number since it contributed valuable qualitative knowledge on professional boundary work in the home setting. Further research is needed on the collaboration between the studied professionals in other countries and in relation to other approaches to home visiting programmes, as well as on boundary work and interprofessional collaboration in the home setting with another compositions of professionals. It should also be valuable to use the combination of interviews and observations in further studies for deeper understanding of interprofessional collaboration in the home setting. Another limitation of this study might be the short period of this study as possible conflicts of interests and values due to different professional and organizational backgrounds might be more visible after a while [40].

## Conclusion

This study contributed knowledge on collaboration in newly created interprofessional teams in the home setting through boundary work as a theoretical background. The professionals in this study emphasized how well they worked together. The boundaries between them were interpreted as maintained, downplayed, and blurred. The informal character of the home setting enabled the collaboration in the teams and contributed to informal professional roles, which shows the merit of home visit programmes. Working for the best of the visited families was a driving force, and the professionals seemed to jointly contribute to a holistic view of the visited families’ support needs, which increased the possibilities of meeting these needs.

### Electronic supplementary material

Below is the link to the electronic supplementary material.

Supplementary Material 1

## Data Availability

The datasets used and analysed during the current study is available from the corresponding author on reasonable request.

## References

[1] Meier N (2015). Collaboration in healthcare through boundary work and boundary objects. Qualitative Sociol Rev.

[2] Global strategy on human resources for health: Workforce. 2030. Geneva: World Health Organization; 2016. https://www.who.int/publications/i/item/9789241511131.

[3] Apesoa-Varano EC (2013). Interprofessional conflict and repair: a study of boundary work in the hospital. Sociol Perspect.

[4] Carmel S (2006). Boundaries obscured and boundaries reinforced: incorporation as a strategy of occupational enhancement for intensive care. Sociol Health Ill.

[5] Hunter B, Segrott J (2014). Renegotiating inter-professional boundaries in maternity care: implementing a clinical pathway for normal labour. Sociol Health Ill.

[6] Liberati AG, Gorli M, Scaratti G (2016). Invisible walls within multidisciplinary teams: disciplinary boundaries and their effects on integrated care. Soc Sci Med.

[7] Powell AE, Davies HTO (2012). The struggle to improve patient care in the face of professional boundaries. Soc Sci Med.

[8] Salhani D, Coulter I (2009). The politics of interprofessional working and the struggle for professional autonomy in nursing. Soc Sci Med.

[9] Barnes W, Bullock A, Chestnutt IG, Cowpe J, Moons K, Warren W (2020). Dental therapists in general dental practice. A literature review and case-study analysis to determine what works, why, how and in what circumstances. Eur J Dent Educ.

[10] Franzén C (2012). Boundary work of dentists in everyday work. Community Dent Oral.

[11] Franzén C (2020). The complexities of boundaries, task claims and professional identity in teamwork: from dentists’ perspective. Professions Professionalism.

[12] Azzi-Lessing L (2011). Home visitation programs: critical issues and future directions. Early Child Res Q.

[13] Finello KM, Terteryan A, Riewerts RJ (2016). Home visiting programs: what the primary care clinician should know. Curr Prob Pediatr Ad.

[14] Larsen A, Broberger E, Petersson P (2016). Complex caring needs without simple solutions: the experience of interprofessional collaboration among staff caring for older people with multimorbidity at home care settings. Scand J Caring Sc.

[15] Vaartio-Rajalin H, Fagerström L (2019). Professional care at home: Patient‐ centredness, interprofessionality and effectivity? A scoping review. Health Soc Care Comm.

[16] Nievar MA, Van Egeren LA, Pollard S (2010). A meat-analysis of home visiting programs: moderators of improvements in maternal behavior. Infant Ment Health J.

[17] Burström B, Marttila A, Kulane A, Lindberg L, Burström K (2017). Practising proportionate universalism: a study protocol of an extended postnatal home visiting programme in a disadvantaged area in Stockholm, Sweden. BMC Health Serv Res.

[18] Swedish Association of Local Authorities and regions. Nära vård för barn och unga. [Integrated care for children and youth]. 2021. skr.se/download/18.5627773817juhilae39e979ef384b0/1642163672815/7585-978-1.pdf. In Swedish.

[19] Axelsson SB, Axelsson R (2009). From territoriality to altruism in interprofessional collaboration and leadership. J Interprof Care.

[20] Williams A (2002). Changing geographies of care: employing the concept of therapeutic landscapes as a framework in examining home space. Soc Sci Med.

[21] Ferguson H (2018). Making home visits: Creativity and the embodied practices of home visiting in social work and child protection. Qual Soc Work.

[22] Hall C, Slembrouck S, Haigh E, Lee A (2010). The management of professional roles during boundary work in child welfare. Int J Soc Welf.

[23] Allen D (2000). Doing occupational demarcation: the ‘boundary work’ of nurse managers in a district general hospital. J Contemp Ethnogr.

[24] Mizrachi N, Shuval J, Gross S (2005). Boundary at work: alternative medicine in biomedical settings. Sociol Health Ill.

[25] Nancarrow SA, Borthwick AM (2005). Dynamic professional boundaries in the healthcare workforce. Sociol Health Ill.

[26] Risan M (2022). Negotiating professional expertise: hybrid educators’ boundary work in the context of higher education-based teacher education. Teach Teach Educ.

[27] Sanders T, Harrison S (2008). Professional legitimacy claims in the multidisciplinary workplace: the case of heart failure care. Sociol Health Ill.

[28] Langley A, Lindberg K, Mørk BE, Nicolini D, Raviola E, Walter L (2019). Boundary work among groups, occupations, and organizations. From cartography to process. Acad Manag Ann.

[29] Alvesson M (2013). The triumph of emptiness: consumption, higher education, and work organization.

[30] Comeau-Vallée M, Langley A (2020). The interplay of inter- and intraprofessional boundary work in multidisciplinary teams. Organ Stud.

[31] National Board of Health and Welfare (2020). Överenskommelserna Om ökad tillgänglighet i barnhälsovården. [Agreement of increased accessibility in child healthcare].

[32] Hinton L, Ryan S, Interviews IC, Pope, Mays N (2020). Qualitative research in health care.

[33] Franzén C (2023). Dental professionals participating in a home visiting programme for first-time parents. Community Dent Health.

[34] Elo S, Kyngäs H (2008). The qualitative content analysis. J Ad Nurs.

[35] Fournier V, Malin N (2000). Boundary work and the (un)making of the professions. Professionalism, boundaries and the workplace.

[36] Schot E, Tummers L, Noordegraaf M (2020). Working on working together. A systematic review on how healthcare professionals contribute to interprofessional collaboration. J Interprof Care.

[37] Brante T. The professional landscape: the historical development of professions in Sweden. Professions Professionalism. 2013;3.

[38] Van Bochove M, Tonkens E, Verplanke L, Roggeveen S (2018). Reconstructing the professional domain: boundary work of professionals and volunteers in the context of social service reform. Curr Sociol.

[39] Allen D (1997). The nursing-medical boundary: a negotiated order?. Sociol Health Ill.

[40] Eriksson A, Bihari Axelsson S, Axelsson R (2012). Collaboration in workplace health promotion: a case study. Int J Workplace Health Manage.

