# Supplementary material for: Supporting first-time parents in their homes: an informal setting enabling interprofessional collaboration
Source: BMC Health Serv Res. 2024 Apr 29;24:545. doi: 10.1186/s12913-024-10949-6 (PMC11059760; doi:10.1186/s12913-024-10949-6)
Supplement: Supplementary file 1 — Supplementary Material 1 [file 12913_2024_10949_MOESM1_ESM.docx]

**The interview guide**

1. Why are you participating in this home visiting programme?
2. What are your expectations for the programme?
3. What do you perceive as the goals of the programme?
4. What opportunities and challenges can you see with the program?
5. Should this extended home visiting programme be offered to all parents or only to some?
6. How do you perceive the collaboration between the professionals in the teams?
7. What is your contribution compared to the other professionals?
8. What expectations do you have for the interprofessional teams?
9. What can the professionals in the teams learn from each other?
10. How do the teams utilize the guidelines for the home visits?
11. Do you have team meetings? If so, could you describe them?
12. Do you feel you receive adequate managerial support and resources?
